# Genome-wide identification and expression analysis of the cryptochromes reveal the *CsCRY1* role under low-light-stress in cucumber

**DOI:** 10.3389/fpls.2024.1371435

**Published:** 2024-04-10

**Authors:** Haishun Cao, Rui Wang, Junhong Zhao, Liangliang Shi, Yuan Huang, Tingquan Wu, Changyuan Zhang

**Affiliations:** ^1^ Institute of Facility Agriculture, Guangdong Academy of Agricultural Sciences, Guangzhou, China; ^2^ National Key Laboratory for Germplasm Innovation & Utilization of Horticultural Crops, College of Horticulture and Forestry Sciences, Huazhong Agricultural University, Wuhan, China

**Keywords:** cryptochromes, cucumber, low-light-stress, photoreceptors, alternative splicing

## Abstract

**Introduction:**

Low-light-stress is a common meteorological disaster that can result in slender seedlings. The photoreceptors play a crucial role in perceiving and regulating plants' tolerance to low-light-stress. However, the low-light-stress tolerance of cucumber has not been effectively evaluated, and the functions of these photoreceptor genes in cucumber, particularly under low-light-stress conditions, are not clear.

**Methods:**

Herein, we evaluated the growth characteristics of cucumber seedlings under various LED light treatment. The low-light-stress tolerant cucumber CR and intolerant cucumber CR were used as plant materials for gene expression analysis, and then the function of *CsCRY1* was analyzed.

**Results:**

The results revealed that light treatment below 40 μmol m^-2^ s^-1^ can quickly and effectively induce low-light-stress response. Then, cucumber CR exhibited remarkable tolerance to low-light-stress was screened. Moreover, a total of 11 photoreceptor genes were identified and evaluated. Among them, the cryptochrome 1 (*CRY1*) had the highest expression level and was only induced in the low-light sensitive cucumber CS. The transcript *CsaV3_3G047490.1* is predicted to encode a previously unknown CsCRY1 protein, which lacks 70 amino acids at its C-terminus due to alternative 5′ splice sites within the final intron of the *CsCRY1* gene.

**Discussion:**

CRY1 is a crucial photoreceptor that plays pivotal roles in regulating plants' tolerance to low-light stress. In this study, we discovered that alternative splicing of *CsCRY1* generates multiple transcripts encoding distinct CsCRY1 protein variants, providing valuable insights for future exploration and utilization of CsCRY1 in cucumber.

## 1 Introduction

Light is one of the most critical environmental factors for living organisms on Earth. Plants can convert light energy into carbohydrates through photosynthesis, as it provides a source of energy for humans (Xu et al., 2015). However, global warming has resulted in an increasing frequency of extreme weather events, particularly for continuously cloudy weather or rainfall (Zhu, 2016). Consequently, low-light-stress has emerged as one of the most significant meteorological disasters worldwide, ultimately impacting photosynthesis, growth, accelerating reproductive development, and leading to lower plant biomass and decreased crop yield and quality (Liu et al., 2014; Sekhar et al., 2019; Casal and Fankhauser, 2023; Li et al., 2023). Over the past few decades, scientists have gradually unraveled crucial molecular mechanisms and signaling pathways by conducting research on photoreceptor genes in Arabidopsis. Nevertheless, there has been limited progress in understanding the mechanism of light signal transduction in cucurbits, particularly cucumbers.

Plants are sessile, so they must constantly adapt to the ever-changing light environment. To achieve this, they employ multiple photoreceptors to respond to wavelengths of light with different intensities ranging from ultraviolet to the far-red regions (Galvão and Fankhauser, 2015). The primary source of low-light-stress is the reduction in sunlight intensity due to continuous cloudy weather, rainfall (Ma et al., 2021), or crowded plant canopies (Casal, 2013). Photoreceptors have the ability to perceive various low-light-stress conditions. In plants, there are four primary types of photoreceptors, including the UVB receptor (280–315 nm UV light), PHYs (600–750 nm red and far-red light), CRYs (350–500 nm blue light), and phototropins (320–500 nm blue light) (De Wit et al., 2016). Among them, PhyB is a critical photoreceptor that can perceive low R: FR of shade light (Casal and Fankhauser, 2023). CRY1 and CRY2 are mainly responsible for sensing the changes in blue light intensity (de Wit et al., 2016; Pedmale et al., 2016). These photoreceptors can regulate gene expression by modulating the activity of transcription factors under low-light-stress, thereby promoting the extension of hypocotyls, stems, and petioles. These responses are collectively referred to as the shade avoidance response (SAR) (Casal, 2013; Pedmale et al., 2016). Currently, the majority of knowledge regarding the activity of photoreceptors was derived from shade-intolerant plants, while their specific roles in low-light-stress tolerant crops remain unexplored.

The cucumber (*Cucumis sativus L*.) is an economically important crop. Low-light-stress will lead to the formation of weak cucumber seedlings with small leaves, long stems, and fewer female flowers (Zhou et al., 2022; Cao et al., 2023; Li et al., 2023). LED supplementary lighting technology has been widely applied to enhance crop growth under low-light-stress, particularly for horticulture crops (Ma et al., 2021; Grishchenko et al., 2022). By utilizing LED supplementary lighting, we can also significantly enhance seedling growth and boost the fruit yield of cucumbers (Song et al., 2019; Gajc-wolska et al., 2021). However, there is limited knowledge about strategies for cucumber response to low-light-stress. In soybean, the cultivars that are sensitive to low-light-stress respond to the decrease in light intensity by significantly increasing the length of their cells. Conversely, in tolerant cultivars, the rate of cell elongation is reduced, yet their photosynthetic efficiency and yield are higher (Lorenzo et al., 2019). Furthermore, it has also been observed that enhancement of *CRY1*-signaling activity can significantly improve yield potential of soybean under low-light-stress conditions (Lyu et al., 2021). The research conducted above has demonstrated that genes in the photoreceptor-signaling pathway play crucial roles in regulating plants’ tolerance to low-light-stress. Nevertheless, the low-light tolerance of cucumber has not been effectively evaluated, and the functions of these photoreceptor genes in cucumber, particularly under low-light-stress conditions, are not clear. In our research, we have established a rapid and efficient evaluation system for assessing cucumber tolerance to low-light-stress and obtained one cucumber material with significantly better tolerance. Additionally, the photoreceptor genes of cucumber were also identified, and a comprehensive analysis was conducted on the role of *CsCRY1* under low-light-stress.

## 2 Materials and methods

### 2.1 Plant materials and LED light treatments

**Figure 1:**
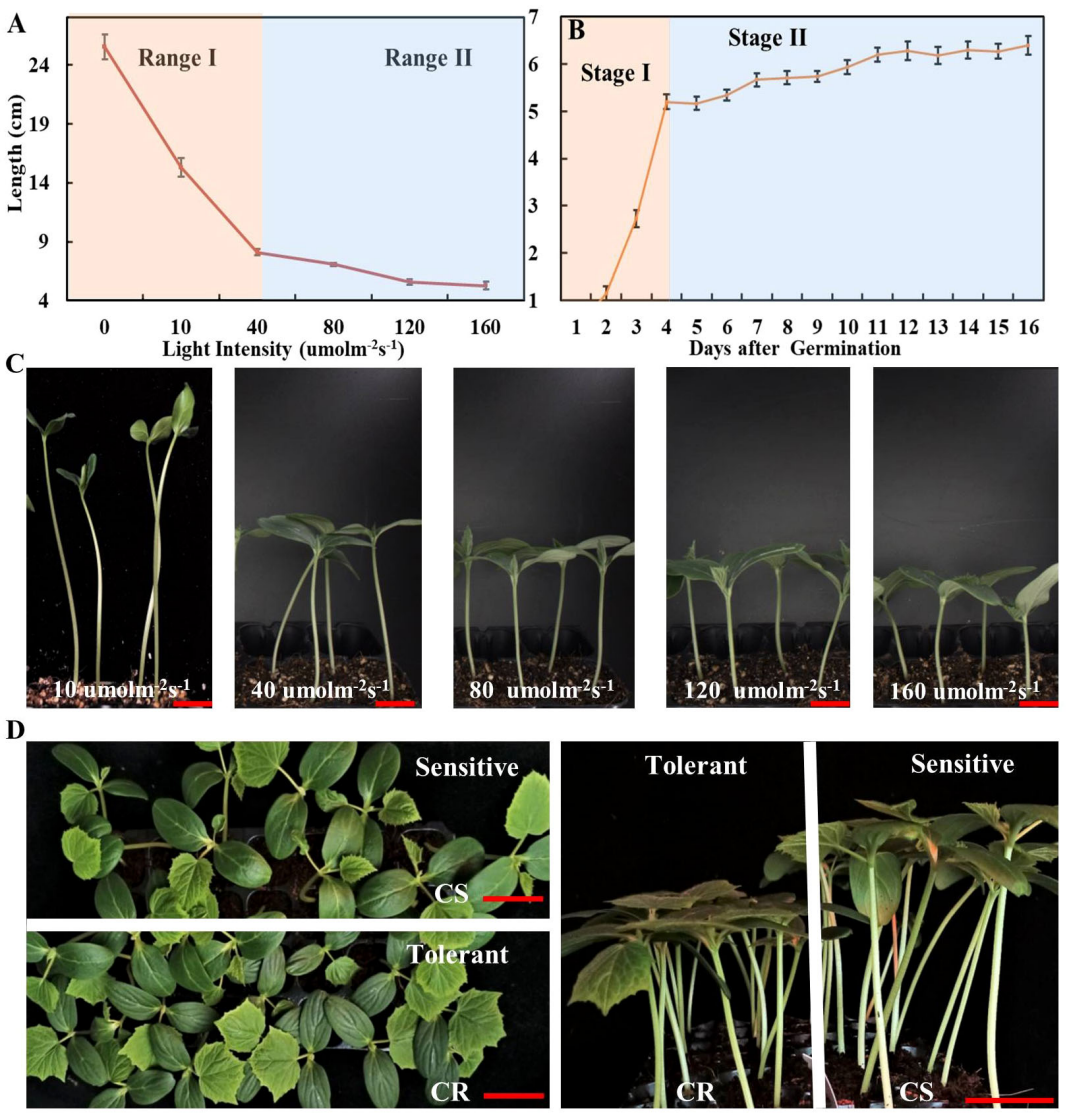
The effect of different light intensities on elongation of cucumber hypocotyl. **(A)** The cucumber hypocotyl length of cucumber seedlings (CS) growth under different light intensities of 10, 40, 80, 120, 160 μmol m^-2^ s^-1^ for one week. **(B)** The length of cucumber hypocotyls (CS) from 0 to 16d under white LED light (160 μmol m^-2^ s^-1^). **(C)** The phenotype of cucumber seedlings (CS) growth under 10, 40, 80, 120, 160 μmol m^-2^ s^-1^ for one week. **(D)** The phenotype of low-light-stress tolerant (CR) and sensitive cucumber (CS) lines under low-light-stress (40 μmol m^-2^ s^-1^) treatment at seedling stage for two weeks. The red scale bar represents a length of 2cm.

The cucumber (*Cucumis sativus*) CS and CR were used as materials in this study, which were preserved at Institute of Facility Agriculture, Guangdong Academy of Agricultural Sciences. CS (EA background) was identified from North South China ecotype cucumber varieties, which is commonly used in modern cucumber breeding with high-quality genome and very rich omics data (Huang et al., 2009; Li et al., 2019). CR (EA background) was identified from the South China ecotype cucumber varieties. Plants were grown in plug trays in a plant incubator that maintained a temperature of (25 ± 1) °C and a 14-hour light/10-hour dark cycle. The relative humidity was 60%-80%. To determine the optimal low-light-stress condition, six distinct white LED light intensity treatments (0, 10, 40, 80, 120, 160 μmol m^-2^ s^-1^) were applied to the CS and CR plants, respectively. The effect of light quality on the growth of cucumber seedlings (CS) was evaluated by four different wavelengths LED light treatment, including 40 μmol m^-2^ s^-1^ white light LED (WL40), 160 μmol m^-2^ s^-1^ white light LED (WL160), 160 μmol m^-2^ s^-1^ LED light composed of blue light (16 μmol m^-2^ s^-1^) and red light (144 μmol m^-2^ s^-1^) (RB91), 160 μmol m^-2^ s^-1^ LED light composed of blue light (144 μmol m^-2^ s^-1^) and red light (16 μmol m^-2^ s^-1^) (RB19) (**Figure 1A**). After two weeks, the growth characteristics of cucumber seedlings were measured.

### 2.2 Identification and characterization of the photoreceptor genes in cucumber

To identify the photoreceptor genes (PHYs, CRYs, UVRs, PHOTs) involved in low-light-stress, the protein sequences of *Arabidopsis thaliana* were retrieved from the The Arabidopsis Information Resource (TAIR) database (https://www.arabidopsis.org/), and these sequences were used as queries to identify the photoreceptors in cucumber by conducting BLASTP searches in the CuGenDBv2 database (http://cucurbitgenomics.org/v2/) (Yu et al., 2023). Then, the Pfam (https://www.ebi.ac.uk/Tools/hmmer/search/phmmer) and MEME suite (http://meme-suite.org/) were utilized to analyze and validate the conserved motifs of these photoreceptor proteins.

### 2.3 Phylogenetic tree, conserved motifs and gene structure analysis of the CRYs

The CRY protein sequences of *Cucumis sativus*, *Citrullus lanatus, Cucumis melo, Cucurbita moschata, Momordica charantia, Lagenaria siceraria, Benincasa hispida, Sechium edule, Luffa cylindrical, Trichosanthes anguina*, and *Cucurbita pepo* were obtained from CuGenDB (http://cucurbitgenomics.org/v2/), and those of *Solanum lycopersicum* were from the Sol Genomics Network (https://solgenomics.net/), and those of *Arabidopsis thaliana* were from TAIR (https://www.arabidopsis.org/). The phylogenetic tree was constructed using MEGA 7 software (Institute of Molecular Evolutionary Genetics, USA). The gene structure of *CsCRY1* was predicted using GSDS 2.0 (http://gsds.gao-lab.org/). The protein length, molecular weight (Mw), and theoretical isoelectric point (pI) of CRY1 were analyzed by the ExPASy ProtParam (https://web.expasy.org/protparam/). The subcellular location of CRY1 was predicted by INSP (http://www.csbio.sjtu.edu.cn/bioinf/INSP/).

### 2.4 Gene expression analysis of *CsCRY1* and the photoreceptor genes from cucurbits

To analyze the gene expression of these photoreceptor genes, we retained the expression data from the Cucurbit Expression Atlas (http://cucurbitgenomics.org/v2/). Among them, tissue expression data of cucumber photoreceptor genes was obtained from the transcriptome atlas of cucumber (PRJNA312872), and tissue expression data of melon was obtained from gene expression atlas of melon (PRJDB6414) (Yano et al., 2018). Tissue expression data of watermelons was obtained from transcriptome profiling of watermelon fruit development (PRJNA543725) (Guo et al., 2019). Then, the heatmaps were constructed using TBtools (Chen et al., 2020).

### 2.5 Alternative splicing (AS) analysis of *CsCRY1* gene from cucumber

Total RNA was extracted from different cucumber tissue of CS (Rt: root, Sm: stem, Tl: tendril, Ap: apical point, Yl: young leaves, Ml: young leaves, Ol: young leaves, Pe: petal, St: stigma, Pi: pistil, Ov: ovary), using an OminiPlant RNA Kit (DNAaseI) from CWBIO (www.cwbio.com). The PrimeScriptTM RT reagent kit (TakaRa, Dalian, China) was using to produce reverse transcribed cDNA. The TSINGKE TSE030 T3 Super PCR Mix was used for RT-PCR assays. We selected *CsACTIN* as the reference gene. All the primer pairs in this study were listed in **Supplementary Table 1**. Three biological replicates were performed. To analyze the expression of *CsCRY1* gene under low-light-stress, RNA-seq of cucumber was conducted using Illumina Novaseq6000 by Gene Denovo Biotechnology Co. (Guangzhou, China). The hypocotyls of CS and CR cucumber were sampled for RNA-seq and RT-PCR after 40 μmol m^-2^ s^-1^ low-light-stress (LL) and 160 μmol m^-2^ s^-1^ normal light (CK) treatments 72h. The software rMATS (version 4.0.1) (http://rnaseq-mats.sourceforge.net/index.html) was used to identify significant AS events with a false discovery rate (FDR) <0.05 (Shen et al., 2014). The *CsCRY1* transcripts structure information was analyzed using IGV-GSAman software (https://gitee.com/CJchen/IGV-sRNA).

### 2.6 Subcellular localization analysis of CsCRY1.1

The full-length CDS of *CsCRY1.1* from CS was amplified by PCR using 2 × High-Fidelity Master Mix (Tsingke, Inc., Beijing, China), and the PCR fagments were inserted in the *KpnI* and *XbaI* site of the pCambia1301-35s-EGFP vector by using ClonExperess II one Step cloning Kits (Vazyme, Piscataway, NJ, United States). The 35S::*CsCRY1.1*-GFP fusion protein was subsequently generated, controlled by the Cauliflower mosaic virus (CaMV) 35S promoter. The vector and the empty vector were each transformed into Agrobacterium strain GV3101. Subsequently, the positive strains were infiltrated into the leaves of tobacco (Nicotiana benthamiana) using the Agrobacterium-mediated transformation method (Sheludko et al., 2007). Finally, laser scanning confocal microscope (CarlZeiss LSM710) was used to take the GFP fluorescence signal pictures.

### 2.7 Statistical analysis

Statistical analysis of the experimental data was performed using SAS statistical package. Results were expressed as means ± standard deviation (SD). Differences in data from treatments were analyzed by one-way ANOVA and the analysis results were corrected with Turkey’s multiple comparison tests at a significance level of P<0.05.

## 3 Results

### 3.1 Low-light-stress can induce rapid elongation of cucumber hypocotyls at the early seedling stage of cucumber

The low-light-stress has a significantly negative impact on the quality of seedlings. However, there is a lack of optimal parameters for evaluating low-light-stress, thus resulting in limited understanding of the mechanism underlying low stress tolerance in cucumber. Therefore, we evaluated the growth characteristics of cucumber (CS) under various light intensities, including 0, 10, 40, 80, 120, and 160 μmol m^-2^ s^-1^, respectively. The hypocotyls elongation was observed to be rapidly promoted, while the leaf size was significantly inhibited by light treatments below 40 μmol m^-2^ s^-1^ (**Figures 1A, C**; **Supplementary Figure 1**). Furthermore, the elongation of cucumber hypocotyl (CS) was evaluated at the whole seedling stage under 160 μmol m^-2^ s^-1^. The results indicated that the elongation process of cucumber hypocotyl can be categorized into two distinct stages: stage I (0-4 days) characterized by rapid elongation of hypocotyl, and stage II characterized by slower elongation of hypocotyl (5-16 days) (**Figure 1B**). Additionally, we also compared the growth of two cultivars (CS and CR) under different light intensities. Firstly, we found that elongation of both material hypocotyls gradually stop after stage I. Secondly, there was a significant difference in the hypocotyls length of CS and CR under 40 μmol m^-2^ s^-1^, while the difference gradually became smaller in other light intensities treatments (**Figure 1D**; **Supplementary Figure 1**). Therefore, we selected 40μmol m^-2^ s^-1^ white LED light treatment for 7 days as the optimal parameters for evaluating low-light-stress. This approach allowed us to quickly and efficiently identify a low-light tolerant cucumber line (CR) and a low-light-sensitive cucumber line (CS) (**Figure 1D**). Notably, CR had a significantly shorter hypocotyl, better photosynthesis capacity, and superior resistance to lodging under low-light-stress compared with CS (**Figure 1D**; **Supplementary Figure 2**).

### 3.2 The decrease in blue light intensity is the primary reason of low-light-stress in cucumber

**Figure 2:**
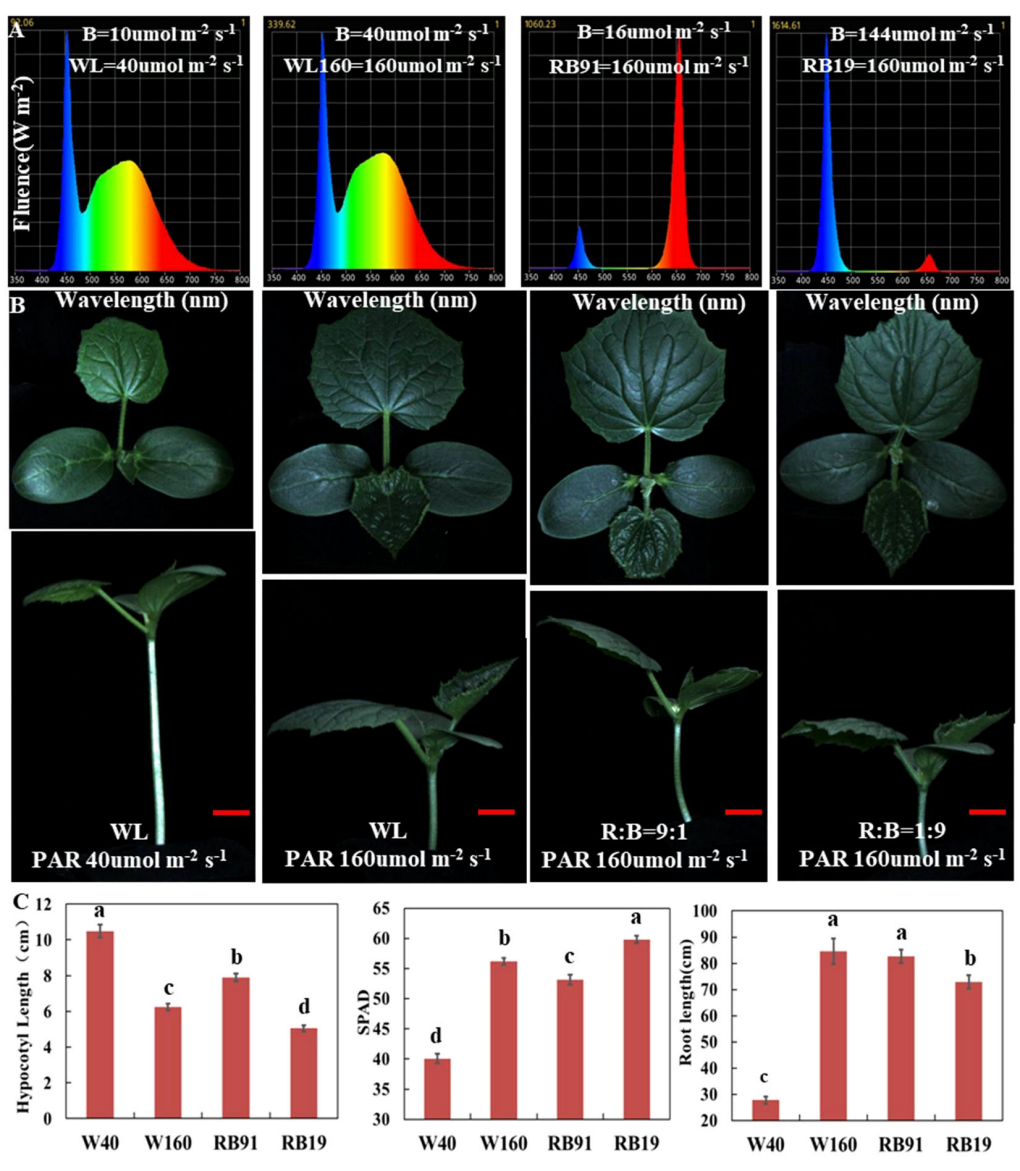
The effect of different light qualities on cucumber seedlings. **(A)** The spectra of four different light combinations. **(B)** Cucumber seedling (CS) phenotypes under four different light combinations. **(C)** The values of hypocotyl length, SPAD and total root length of cucumber seedlings grown for two weeks under four light combinations. Here, the low case letters indicate significant differences at P < 0.05 by the least significant difference test. The red scale bar represents a length of 2cm.

To assess the effect of different light qualities under low-light-stress, we cultivated cucumber seedlings for two weeks under four LED conditions with different intensity of blue and red light: WL40 (40 μmol m^-2^ s^-1^ total light intensity, containing 10 μmol m^-2^ s^-1^ blue light intensity), WL160 (160 μmol m^-2^ s^-1^ total light intensity, containing 40 μmol m^-2^ s^-1^ blue light intensity), RB91 (160 μmol m^-2^ s^-1^ total light intensity, containing 16 μmol m^-2^ s^-1^ blue light intensity) and RB19 (160 μmol m^-2^ s^-1^ total light intensity, containing 144 μmol m^-2^ s^-1^ blue light intensity) (**Figure 2A**). The results revealed that under the WL40 condition, the hypocotyl length was the longest, whereas the SPAD value and total root length were the smallest. When the light intensity was the same, the hypocotyl of treatment RB91 was the longest, followed by treatment WL160, and the shortest was treatment RB19. The results indicated that the hypocotyl length was totally negatively correlated with the intensity of blue light (**Figures 2B, C**). Additionally, our findings revealed that CR exhibited significantly shorter hypocotyls compared to CS when exposed to blue light treatment. However, no significant difference was observed in hypocotyl length between CS and CR under red light treatment (**Supplementary Figure 3**). Furthermore, the supplementary LED experiment demonstrated that the elongation of CS hypocotyls can also be most effectively inhibited by supplement of blue light, whereas red light exhibited the least effective inhibitory effect (**Supplementary Figure 4**). The research findings indicated that the reduction in blue light intensity is the primary factor responsible for inducing low-light-stress in cucumber.

### 3.3 CsaV3_3G047490 is highest expressed photoreceptor genes in cucumber

**Figure 3:**
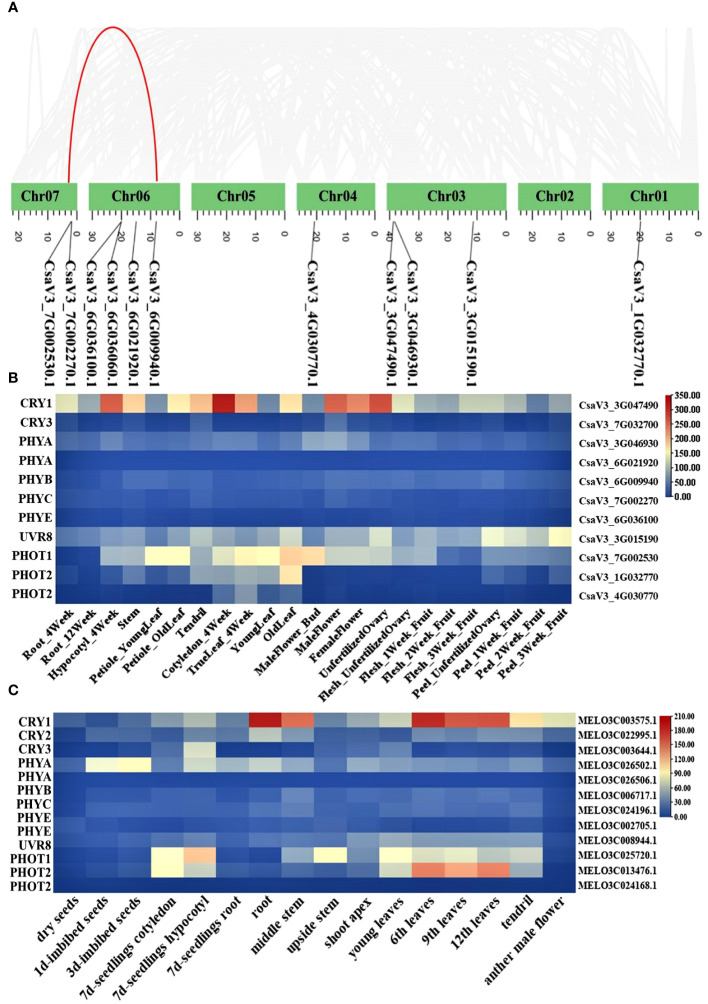
Chromosome location of photoreceptor genes in cucumber **(A)**; Heat map of photoreceptor genes in different tissue from cucumber **(B)** and melon **(C)**.

Photoreceptors play a critical role in regulating low-light-stress tolerance of plants. However, in cucurbit crops, photoreceptor genes have not been clearly explained in detail. Here, we identified the major classes of photoreceptors (*CRYs, UVRs, PHYs, PHOTs*) and analyzed their expression patterns in different cucurbit crops. A total of 11 and 13 photoreceptor genes were identified in cucumber and melon, respectively. The gene ID information of these factors was shown in **Supplementary Table 2**. Chromosome mapping results revealed that the 11 photoreceptor genes in cucumber were each mapped to five distinct chromosomes. In the photoreceptors, the *PHYA* and *PHOT2* genes have an additional copy in almost all cucurbit crops. Furthermore, a segmental duplication gene pair (PHOT2) was identified through syntenic analysis (**Figure 3A**). It is well-recognized that analyzing the expression of these genes across different tissues is pivotal in deciphering their function. The heatmaps, created using expression data from the Cucurbit Expression Atlas, revealed that *CsaV3_3G047490.1* consistently exhibited a higher expression level than other photoreceptor genes in various cucurbit crops, including cucumber, melon, watermelon, and bitter melon (**Figures 3B, C**; **Supplementary Figure 5**). Additionally, CRY1 was also highly expressed in the hypocotyls of cucumber seedlings (**Figure 3B**).

### 3.4 *CsaV3_3G047490.1* belongs to the cryptochrome blue light receptors and was named *CsCRY1*

**Figure 4:**
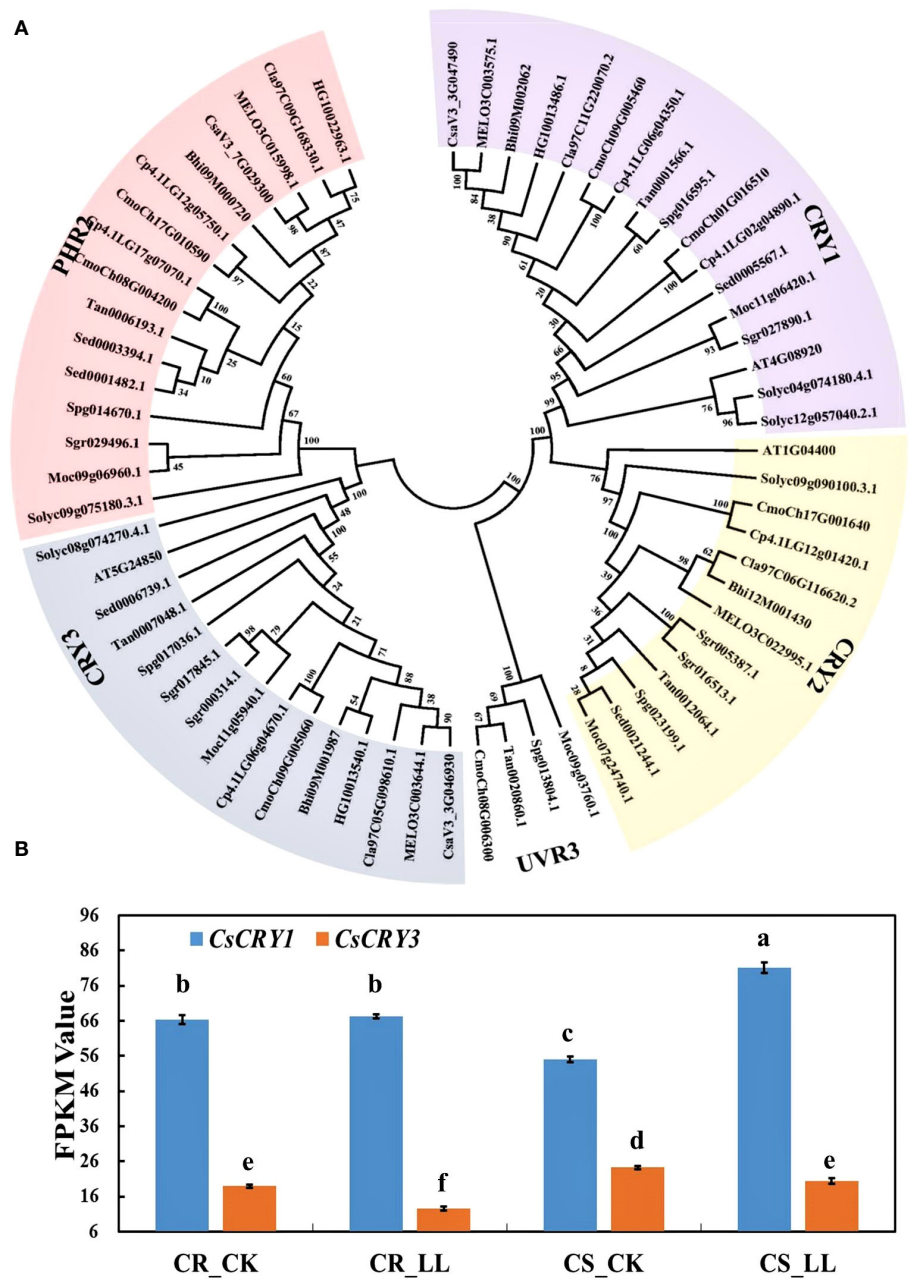
The phylogenetic tree and expression analysis of *CsaCRYs* gene in cucumber. **(A)** The phylogenetic tree and subgroup classifications of CRY proteins in cucumber, melon, watermelon, bitter melon, wax gourd, bottle gourd, pumpkin, tomato, and Arabidopsis. **(B)** Expression profiles of *CsCRY1* and *CsCRY3* genes in low-light-stress -tolerant (CR) and light-stress-sensitive (CS) cucumber cultivars under 40μmol m^-2^ s^-1^ low-light-stress treatment (LL) and 160μmol m^-2^ s^-1^ control (CK) treatment. Here, the low case letters indicate significant differences at P < 0.05 by the least significant difference test.

CsaV3_3G047490 was annotated as the cryptochrome blue light receptors in the cucumber genome. To further identify the *CsCRY* genes, we used 3 CRYs (AtCRY1, AtCRY2 and AtCRY3) protein sequences from Arabidopsis as queries to carry out a Blastp search. As a result, only two *CRYs* genes were identified in the Chinese Long v3 genome of cucumber. To investigate the evolutionary relationships among the CRY proteins in cucurbit crops, a phylogenetic tree was constructed using MEGA 7. All the CRYs proteins in cucurbits crops could be divided into three subfamilies: CRY1, CRY2, and CRY3 according to the classification of CRYs in Arabidopsis. The CRY1 subfamily has the highest number of members, with a total of 17. Conversely, the CRY2 subfamily is the smallest with only 13 members (**Figure 4A**).

The physical and chemical properties of CsCRY1 and CsCRY3 were shown in **Table 1**. The amino acid sequence lengths of CsCRY1 and CsaCRY3 are 613 amino acids and 592 amino acids, respectively, while their pI values are 5.46 and 9.4. Both *CRYs* genes are mapped to almost the same region of chromosome 3 (**Figure 3A**). Interestingly, the *CRY2* gene is absent in the cucumber 9930 genome, but not in other cucurbit crops such as melon, watermelon, pumpkin, and bitter melon (**Table 1**; **Supplementary Table 2**). Therefore, we identified the cucumber *CRY2* gene in the remaining four reference cucumber genomes within CuGenDBv2 database. Interestingly, we identified a completely intact *CRY2* gene (636aa) in *Cucumis hystrix var* (2n = 2x = 24), a wild cucumber species that can hybridize with cultivated cucumber varieties (*C. sativus* L., 2n =2x = 14). The *CRY2* is incomplete (229 amino acids) in the genome of wild/semi-wild varieties *Cucumis sativus* var. *hardwickii* cv. *PI 183967.* All cultivated cucumbers (*Cucumis sativus L.* var. *sativus* cv. *Chinese Long, Cucumis sativus L.* var. *sativus* cv. *Gy14, and Cucumis sativus L.* var. *sativus cv B10*) lack the *CRY2* gene (**Supplementary Table 3**). The findings suggest that the *CsCRY2* gene has been explicitly lost over a long evolution from wild to cultivated varieties.

**Table 1:** Characteristics of *CsCRYs* genes and their annotated information in *Cucumis sativus L*.

Gene ID	Gene name	Strand	Gene position	CDS/bp	Proten/aa	MW/kD	pI	Subcellar location
CsaV3_3 G047490	*CsCRY1*	+	38763751-38768676	1839	612	69.366	5.46	Nucleus
CsaV3_3 G046930	*CsCRY3*	–	38309641-38315065	1779	592	67.434	9.4	Nucleus

### 3.5 The expression of *CsCRY1 was* induced by low-light-stress

To investigate the responses of the *CsCRY* genes to low stress, RNA-seq analysis was conducted on tolerant and sensitive cucumber hypocotyls. The hypocotyls were sampled after 72 hours of treatment under 40μmol m^-2^ s^-1^ (LL) and 160μmol m^-2^ s^-1^ (CK) white light LED. During this period, the hypocotyl length of the tolerant cucumber CR was significantly shorter than that of the sensitive cucumber CS under low-light-stress (**Supplementary Figure 6**). The expression result of *CsCRY1* and *CsCRY3* indicated that the expression level of *CsCRY1* was much higher than *CsCRY3*. Additionally, the expression of *CsCRY3* was significantly depressed by low-light-stress treatment in both tolerant and sensitive cucumber material. However, only in the sensitive cucumber material, the expression of *CsCRY1* was significantly induced by low-light-stress (**Figure 4B**). Therefore, the above results indicated *CsCRY1* may play a critical role under low-light-stress.

### 3.6 Cucumber CsCRY1 protein lost the last 70 aa in the C-terminal

**Figure 5:**
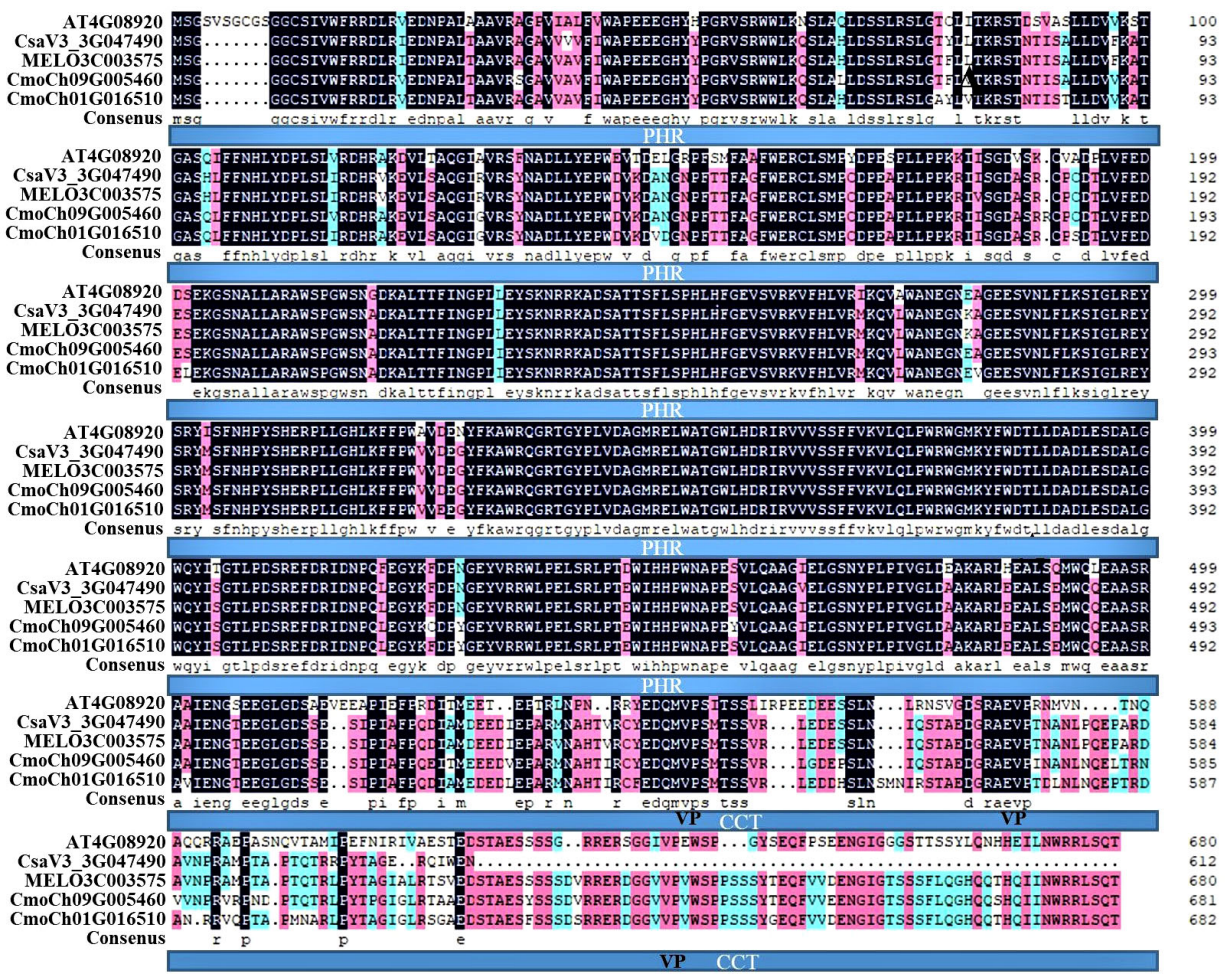
Multiple protein sequence alignment of cucumber CsCRY1. CRY1 protein sequences included AT4G08920 from Arabidopsis, CsaV3_3G047490.1 from *Cucumis sativus L.* var. *sativus* cv. *Chinese Long*, MELO3C003575.2.1 from *Cucumis melo* cv. *DHL92*, CmoCh09G005460, and CmoCh01G016510 from *Cucurbita moschata* var. *Rifu*.

To further understand the functions of CsCRY1, we aligned the amino acid of CRY1 protein from various cucurbit species. Our findings indicated that CRY1 is highly conserved, particularly in its N-terminal PHR domain. However, the length of all CRY1 proteins from various crops is almost 681 amino acids, except for cucumber (**Supplementary Table 4**). The C-terminal of CCT domain in CsCRY1 of cucumber was short 70 aa (**Figure 5**). Then, we cloned the CsCRY1 gene and verified the presence of CsCRY1 mRNA in the cucumber genome, which is capable of encoding a short CsCRY1 protein. It is well-established that both *CRY1* and *CRY2* regulate low-light-stress, albeit a relatively minor role for *CRY2* compared with that of *CRY1 in* Arabidopsis. However, *CsCRY2* was absent in cucumber. The absence of the *CsCRY2* gene in cultivated cucumber leads to a significant role for CsCRY1 in low-light-stress conditions.

### 3.7 AS leads to form one special transcript that encoded CsCRY1.1 protein lost 70 aa

**Figure 6:**
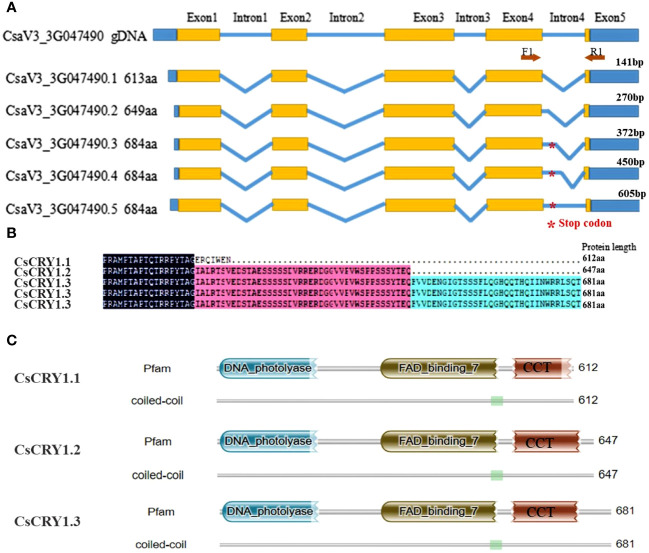
The whole-length transcripts structure of cucumber *CsCRY1*. **(A)** five kinds of 5’SS events in intron 4, * represent the stop codon at the middle of intron 4. **(B)** Alignment of the spliced protein encoding by five splicing variants. Here, only the C-terminal protein was shown. **(C)** The conserved motif analysis of the spliced protein using HMM.

To further elucidate the distinct features of cucumber *CsCRY1*, we have successfully obtained 34 transcripts including 15 full-length transcripts of *CsCRY1* through single-molecule long-read sequencing in cucumber CS (**Supplementary Figure 7**). By utilizing the IGV-GSAman software to align these *CsCRY1* transcripts to the cucumber genome, we discovered that the pre-mRNA of *CsCRY1* undergoes alternative splicing (AS). Additionally, alternative 5′ splice sites (5’SS) in the last intron was the major AS events. Interestingly, there were five kinds of 5’SS events in intron four, one total intron retention event, three partial intron retention events, and one total intron splicing event. We predicted the open reading frames (ORFs) of these *CsCRY1* transcripts. The findings indicated that intron 4 contains a crucial coding sequence, with the stop codon positioned at the midpoint of intron four. Therefore, the five splice variants can be translated into three kinds of splice proteins with different lengths of C-terminal domains (**Figure 6A**). The transcripts *CsaV3_3G047490.3*, *CsaV3_3G047490.4*, and *CsaV3_3G047490.5* encode the full-length protein of CRY1, designated as CsCRY1.3. The transcripts *CsaV3_3G047490.1* and *CsaV3_3G047490.2* encode two different truncated C-terminal CRY1 proteins, named CsCRY1.1 and CsCRY1.2 (**Figure 6B**). We analyzed the conserved domains of these spliced proteins by HMMER. All the spliced protein contains an intact DNA photolyase domain and FAD binding domain. However, only *CsaV3_3G047490.1* contained an impaired CCT domain (**Figure 6C**). Moreover, we did not find the 5’SS of *CRY1* in other species including melon, pumpkin, Arabidopsis, etc. (**Supplementary Figure 8**). Therefore, we concluded that 55’SS of *CsCRY1* is responsible for the formation of various transcripts with diverse coding capabilities in cucumber. However, the precise role of *CsCRY1* remains elusive.

### 3.8 Analysis of 5’SS of *CsCRY1* in cucumber different tissues and stress treatment

**Figure 7:**
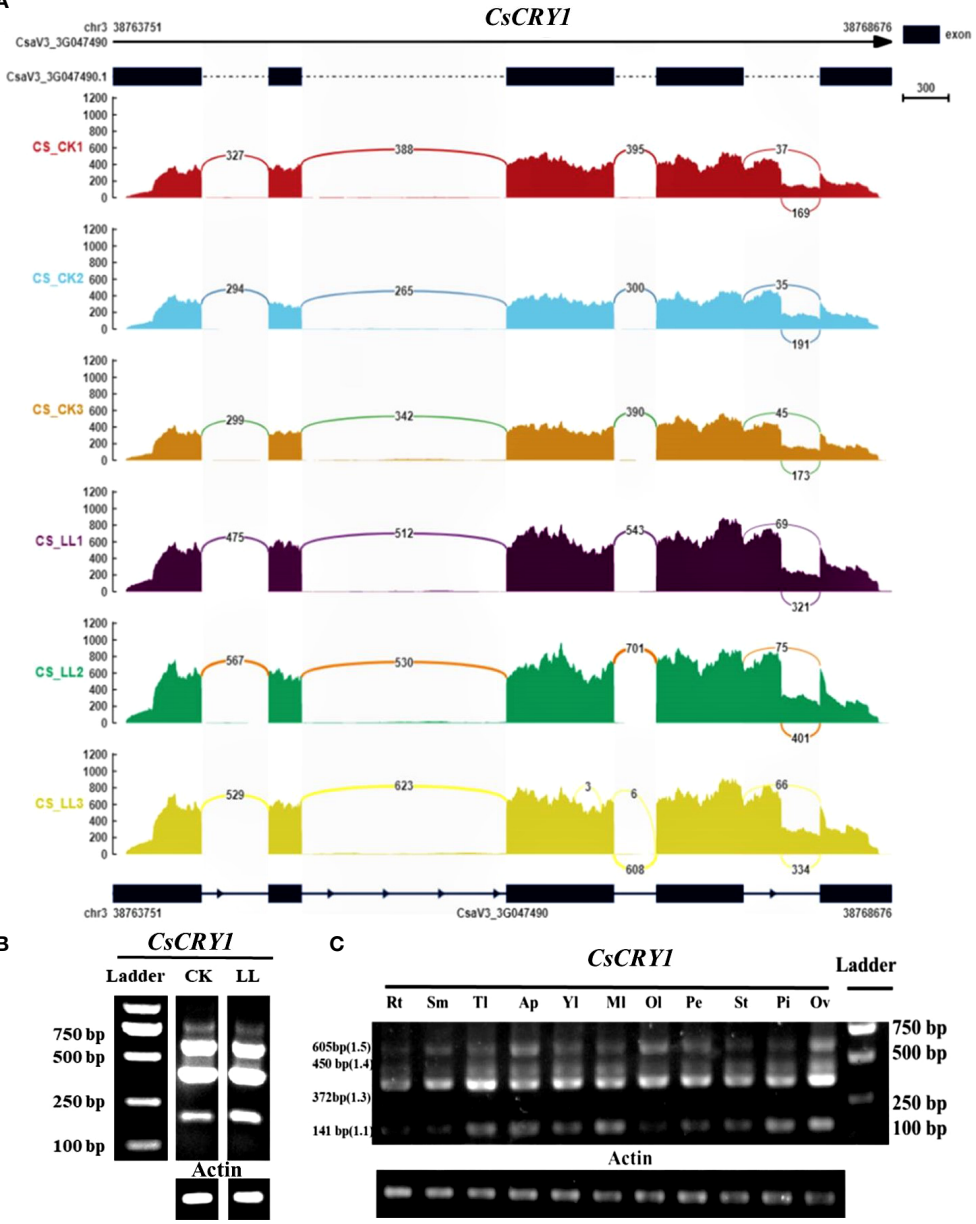
The transcripts read mapping **(A)** and RT-PCR results **(B)** of *CsCRY1* under control and low-light-stress condition, RT-PCR results of *CsCRY1* from different tissues **(C)** (Rt, root; Sm, stem; Tl, tendril; Ap, Apical point; Yl, young leaves; Ml, young leaves; Ol, young leaves; Pe, petal; St, stigma; Pi, pistil; Ov, ovary). CS was used as material. The PCR product size of splicing variant *CsaV3_3G047490.1* was 141bp, the PCR product size of splicing variant *CsaV3_3G047490.1* was 270bp, The PCR product size of splicing variant *CsaV3_3G047490.3* was 372bp, The PCR product size of splicing variant *CsaV3_3G047490.4* was 450bp, and The PCR product size of splicing variant *CsaV3_3G047490.5* was 605bp.

To further validate the AS of CsCRY1, we conducted a transcriptome analysis of both CR and CS cucumber hypocotyls following 72 hours of exposure to low-light-stress (40 μmol m^-2^ s^-1^) and normal light (160 μmol m^-2^ s^-1^) treatment. The results reaffirmed the presence of a 5’SS in the last intron of *CsCRY1* in both CR and CS (**Figure 7A**; **Supplementary Figure 9**). To unravel the expression pattern of *CsCRY1* splicing variants in different tissues, we designed a pair of the primer crossing the intron four to perform RT-PCR assay. The results revealed that there were four spliced transcripts identified in almost all the samples. Among them, the expression level of *CsaV3_3G047490.3* was the highest, followed by *CsaV3_3G047490.4* and *CsaV3_3G047490.1*. In contrast, the expression of *CsaV3_3G047490.5* was the lowest, while no expression of *CsaV3_3G047490.2* was detected (**Figures 7B, C**). Furthermore, all four transcripts were present in hypocotyls under low-light-stress conditions. However, we only found that the expression of *CsaV3_3G047490.1* was induced by low-light-stress, and the RNA-seq analysis confirmed these findings (**Figure 7**; **Supplementary Figure 9**).

**Figure 8:**
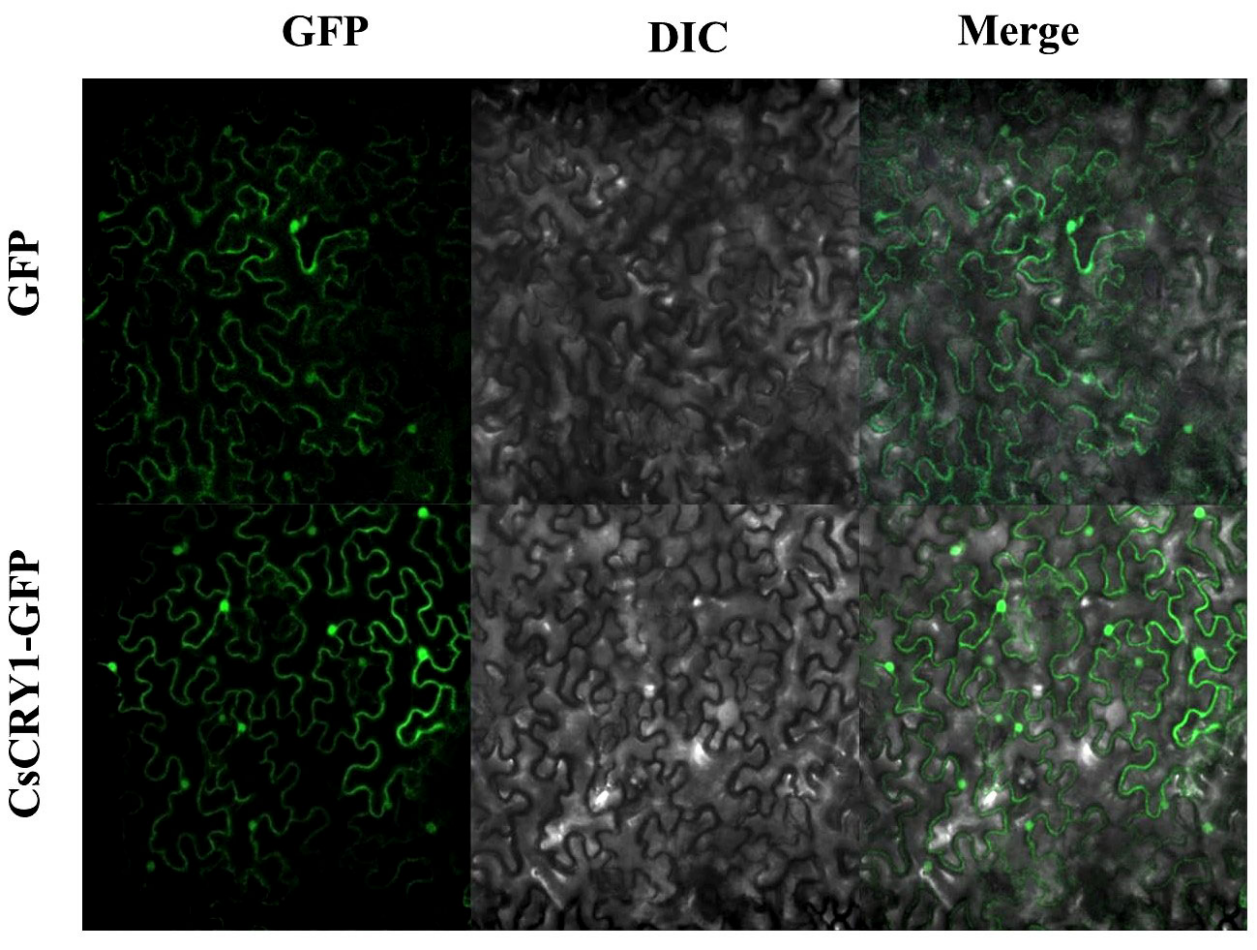
Subcellular localization of CsCRY1.1 CsCRY1.1-GFP fusion proteins and GFP were transiently expressed in tobacco leaves under control of the CaMV 35S promoter and observed under a laser scanning confocal microscope, GFP images, DAPI stained images, differential interference contrast images (DIC), and merged images were taken.

The CsCRY1.1 protein, a newly identified blue light receptor in plants, was cloned and its subcellular localization was evaluated. Here, we found that the subcellular localization of CsCRY1.1 was same to GFP. The fluorescence signal was widely observed in both the cytoplasm and nucleus (**Figure 8**). The result indicated that loss of the last valine-proline-containing (VP) motif did not change subcellular localization of CsCRY1.1. But, whether CsCRY1.1 without the last VP motif can interact with the COP1/SPA complex is still unknown.

## 4 Discussion

Recently, low-light-stress frequently occurs worldwide. It has the potential to induce excessive growth in stems, lead to a decrease in female flowers, and consequently, reduce fruit production (Zhou et al., 2022; Cao et al., 2023; Li et al., 2023). Cucumber is an important crop cultivated in facilities, and also very sensitive to low-light-stress. CS was commonly used in China modern cucumber breeding with high-quality genome and very rich omics data (Huang et al., 2009; Li et al., 2019). Utilizing CS, we established a screening system for low-light-stress tolerance, revealing that blue light and photoreceptor gene *CsCRY1* may play significant roles in this process. The photoreceptor genes play a crucial role in regulating flowering, growth, and production under low-light-stress, particularly for CRYs and PHYs (Yu et al., 2010; Casal and Fankhauser, 2023). There are three *CRYs*, but *CRY3* does not regulate hypocotyl elongation under low-light-stress (Yu et al., 2010). *CRY1* and *CRY2* can interact with PIFs to regulate hypocotyl growth in a limited blue light environment (de Wit et al., 2016; Pedmale et al., 2016). The cry1cry2 double mutant, as mentioned, displayed the most remarkable long hypocotyl phenotype compared to the single mutant cry1 or cry2. Additionally, in comparison to *CRY2*, *CRY1* played a more crucial role in inhibiting hypocotyl growth. Overexpression of *AtCRY1* can result in the failure of hypocotyl elongation under low-light-stress. By elevating the CRY1-signaling activity in soybean, its yield can be significantly enhanced under low-light conditions (Lyu et al., 2021). The findings above suggested that *CRYs* genes played a crucial role in regulating plant tolerance to low-light-stress.

Most plants possess two well-characterized cryptochromes, *CRY1* and *CRY2* (Wang et al., 2021). However, we found that the *CRY2* gene has been lost specifically over a long period of evolution from wild to cultivated cucumber varieties. *CRY2* can also regulate photomorphogenesis, albeit playing a relatively minor role compared with that of *CRY1. CRY2* primarily mediated blue-light photoperiodic control of floral initiation, and the cry2 mutant exhibits a late-flowering phenotype (Yu et al., 2010; Liu et al., 2018). Arabidopsis is a long-day (LD) flowering plant (Wang et al., 2016). However, Xishuangbanna (XIS) cucumber, a semi-wild cucumber, is strictly short-day plants, while cultivated cucumber is day-neutral plants. The expression of *FLOWERING LOCUS T* (*FT*) gene under LD and SD conditions is responsible for regulating short-day flowering in the XIS cucumber (Song et al., 2023). In Arabidopsis*, CRY2* can promote *FT* gene expression by suppressing the degradation of the CO (CONSTANS) protein and activating CIB1 (CRY2-interacting bHLH1) (Liu et al., 2018). *CRY2* is highly conserved, and it’s possible that cucumber CRY2 can also regulate the *FT* gene expression under various photoperiod conditions. Therefore, we can infer that the loss of *CRY2* could be crucial for cultivated cucumbers to become day-neutral plants.

In cucumber, only one *CRY1* gene was identified, and we found that *CRY1* was also the highest-expressed photoreceptor gene. Additionally, the blue light most effectively inhibits the elongation of hypocotyls (**Figure 2**; **Supplementary Figure 1**). The findings suggest that *CsCRY1* plays a crucial role in cucumber’s response to low-light-stress. The CRY1 protein, a highly conserved blue light receptor, possesses an N-terminal domain (PHR) that has evolved from DNA photolyase and a CCT domain (Yu et al., 2010; Wang et al., 2021). The CCT domain can relay the blue light signals perceived by PHR domain, and subsequently interact with the WD40 domain of constitutive photomorphogenic1 (COP1) and suppressor of phya-105 (SPA) in a blue light-specific manner (Yu et al., 2007). Overexpression of CCT1 or CCT2 fused to β-glucuronidase (GUS) resulted in a constitutive photomorphogenic phenotype (shorted hypocotyls, enhanced anthocyanin production and early flowering phenotype) (Yang et al., 2000). Moreover, the homodimerization of CRY1 is crucial for the function of CCT (Sang et al., 2005). Hence, CCT domain of CRY1 is very important.

The CCT domain of CsCRY1 in cucumber was not intact, lacking the last VP motif. In contrast, AtCRY1 carried 3 VP motifs in its CCT domain, while CRY2 only carried 1 VP motif. COP1-SPAs complex usually interacted with the proteins possessing VP motifs (Holm et al., 2001). In Arabidopsis, it was demonstrated that the VP motif in CRY2 was necessary for the CRY2–COP1 interaction (Zuo et al., 2011; Ponnu et al., 2019). Moreover, GFP-CRY2 fully complemented the elongated hypocotyl phenotype of cry1 cry2 in blue light. In contrast, The GFP-CRY2-VP with a mutated VP motif failed to complement the cry1 cry2 mutant phenotype (Ponnu et al., 2019). Therefore, the VP motif plays a crucial role in the CRYs’ function. Furthermore, it was reported that AtCRY2 is exclusively localized in the nucleus. On the other hand, AtCRY1 is present in both the nucleus and cytoplasm, regardless of light or dark conditions, without experiencing a significant alteration in its relative subcellular concentration (Yu et al., 2010). Additionally, it is the nuclear, rather than cytoplasmic, form of CRY1 that effectively inhibits growth (Wu and Spalding, 2007). Here, we found that the subcellular localization of CsCRY1.1 was same to AtCRY1 and GFP. The fluorescence signal was widely observed in both the cytoplasm and nucleus (**Figure 8**). The result indicated that loss of the last VP motif did not change subcellular localization of CsCRY1.1. However, it remains unclear whether CsCRY1.1, which lacks the VP motif, can interact with the COP1/SPA complex.

Through AS, one gene locus can be used to produce multiple crucial mRNA splicing variants with different coding sequences by the spliceosome for coping with fluctuating light environments in eukaryotes (Kathare and Huq, 2021). In Arabidopsis, it was reported almost 85% of genes were multiexon, and 70% of them were alternatively spliced (Martín et al., 2021). Among them, several crucial factors in the light signaling pathway, including *PIF3*, *PIF6*, *ELONGATED HYPOCOTYL5* (*HY5*), and *SPA3*, undergo alternative splicing (Kathare and Huq, 2021). In this study, we present a novel alternative splicing (AS) event in cucumber, specifically the 5’SS of CsCRY1. This AS event results in the production of a unique and distinct CsCRY1.1 protein variant that lacks the VP motif found at the protein’s C-terminus. This novel CsCRY1.1 protein variant may play a pivotal role in the adaptive response of cucumber, a specialized crop, to low-light-stress.

## 5 Conclusions

In this study, we establish an efficient system for evaluation of cucumber low-light-stress tolerance. One cucumber material CR was screened out. Furthermore, a total of 11 photoreceptor genes (*CRYs*, *UVRs*, *PHYs*, *PHOTs*) were identified in cucumber, including 2 *CRYs* genes, *CsCRY1* and *CsCRY3*. Transcriptome data revealed that *CsCRY1* had the highest expression level and was induced expression. Additionally, blue light can most effectively inhibit hypocotyl elongation. CsCRY1 was lost 70 aa in CCT domain. Through single-molecule long-read sequencing and transcriptome analysis, we also found that *CsCRY1* suffer 5’SS in the last intron leading to forming five splicing variants. Among them, *CsaV3_3G047490.1* was predicted to encode the CsCRY1 protein in the reference genome. And its expression was also induced by low-light-stress, which was confirmed by RNA-seq and RT-PCR experiment. Taken together, these results provided crucial information for further research and utilization of *CsCRY1* in cucumber.

## Data availability statement

The datasets presented in this study can be found in National Genomics Data Center (NGDC) repositories, accession numbers (PRJCA024948 and CRA015726). 

## Author contributions

HC: Formal analysis, Investigation, Methodology, Writing – original draft. RW: Methodology, Resources, Writing – review & editing. JZ: Investigation, Methodology, Software, Writing – review & editing. LS: Formal analysis, Resources, Supervision, Writing – review & editing. YH: Investigation, Supervision, Writing – review & editing. TW: Data curation, Formal analysis, Methodology, Resources, Supervision, Writing – review & editing. CZ: Data curation, Formal analysis, Funding acquisition, Resources, Writing – review & editing.
