# Supplementary material for: Genome-wide identification and expression analysis of the cryptochromes reveal the CsCRY1 role under low-light-stress in cucumber
Source: Front Plant Sci. 2024 Apr 10;15:1371435. doi: 10.3389/fpls.2024.1371435 (PMC11040678; doi:10.3389/fpls.2024.1371435)
Supplement: Supplementary file 1 [file DataSheet_1.docx]

| **Table S1 Primer sequences used for expression analysis in this study**   \| Gene name \| Forward primer (5’-3’) \| Reverse primer (5’-3’) \| \| --- \| --- \| --- \| \| Cs-*actin* \| CGTGTTGCTCCCGAAGAACA \| ACCATCACCAGAATCCAGCA \| \| *CRY1* RT-PCR \| CAACTGCTCCGACACAGACT \| TGTTCACCATCTGGCTGACC \| \| *CRY1* CDS \| ATGTCAGGTGGCGGTTGTAG \| CTAGTTTTCCCATATTTGCCTCTCAC \| |  |  |  |  |  |
| --- | --- | --- | --- | --- | --- | --- | --- | --- | --- | --- | --- | --- | --- | --- | --- | --- | --- |

**Table S2 The photoreceptor genes information in different plants**

| **Gene Name** | ***Arabidopsis thaliana*** | ***Solanum lycopersicum*** | ***Citrullus lanatus*** | ***Cucumis sativus*** | ***Cucumis melo*** | ***Lagenaria siceraria*** | ***Cucurbita moschata*** |
| --- | --- | --- | --- | --- | --- | --- | --- |
| *CRY1* | AT4G08920 | Solyc04g074180 Solyc12g057040 | Cla97C11G220070 | CsaV3_3G047490 | MELO3C003575 | Lsi04G022320 | CmoCh09G005460 CmoCh01G016510 |
| *CRY2* | AT1G04400 | Solyc09g090100 | Cla97C06G116630 | NA | MELO3C022995 MELO3C018340 | NA | CmoCh17G001640 |
| *CRY3* | AT5G24850 | Solyc08g074270 | Cla97C05G098610 | CsaV3_3G046930 | MELO3C003644 | Lsi04G021800 | CmoCh09G005060 |
| *PHYA* | AT1G09570 | Solyc10g044670 | Cla97C08G151850 Cla97C08G151880 | CsaV3_6G036100 CsaV3_6G036060 | MELO3C026502 MELO3C026506 | Lsi08G007550 Lsi08G007580 | CmoCh05G009670 CmoCh12G006390 |
| *PHYB* | AT2G18790 | Solyc01g059870 Solyc05g053410 | Cla97C05G088180 | CsaV3_3G015190 | MELO3C006717 | Lsi05G014200 | CmoCh14G016190 CmoCh06G015600 |
| *PHYC* | AT5G35840 | Solyc07g045480 | Cla97C02G029370 | CsaV3_7G002530 | MELO3C024196 | Lsi11G013450 | CmoCh19G008690 |
| *PHYD* | AT4G16250 | NA | NA | NA | NA | NA | NA |
| *PHYE* | AT4G18130 | Solyc02g071260 | Cla97C03G058420 | CsaV3_1G032770 | MELO3C002705 | Lsi02G008570 | CmoCh13G005960 CmoCh18G004270 |
| *UVR8* | AT5G63860 | NA | Cla97C04G069610 | CsaV3_4G030770 | MELO3C008944 | Lsi01G019870 | CmoCh07G013100 |
| *PHOT1* | AT3G45780 | Solyc11g072710 | Cla97C02G046720 | CsaV3_6G021920 | MELO3C025720 | Lsi10G013210 | CmoCh02G008530 CmoCh20G004870 |
| *PHOT2* | AT5G58140 | Solyc01g097770 | Cla97C02G036540 Cla97C02G029650 | CsaV3_6G009940 CsaV3_7G002270 | MELO3C013476 MELO3C024168 | Lsi10G000180 Lsi11G013200 | CmoCh20G006850 CmoCh02G003040 CmoCh19G008470 |

**Table S3 The *CRY2* gene information in different plants**

| **Gene ID** | **Gene name** | **Strand** | **Gene position** | **CDS/bp** | **Proten/aa** | **Chr** | **Species** |
| --- | --- | --- | --- | --- | --- | --- | --- |
| AT1G04400 | *AtCRY2* | _ | 1185512-1188696 | 1839 | 612 | 1 | *Arabidopsis thaliana* |
| NA | *CsCRY2* | + | NA | NA | NA | NA | *Cucumis sativus L. var. sativus cv. Chinese Long* |
| NA | *CsCRY2* | + | NA | NA | NA | NA | *Cucumis sativus L. var. sativus cv. Gy14* |
| NA | *CsCRY2* | + | NA | NA | NA | NA | *Cucumis sativus L. var. sativus cv B10* |
| CSPI02G10340 | *CsCRY2* | + | 10411672-10415331 | 687 | 228 | 2 | *Cucumis sativus var. hardwickii cv. PI 183967* |
| Chy5G093690 | *CsCRY2* | + | 3243394-3247549 | 1902 | 633 | 5 | *Cucumis hystrix (Cucumber (hystrix)* |
| MELO3C022995 | *CmCRY2.1* | _ | 5666014 - 5670839 | 1902 | 633 | 5 | *Cucumis melo cv. DHL92* |
| MELO3C018340 | *CmCRY2.2* | + | 18464050 - 18466637 | 327 | 108 | 10 | *Cucumis melo cv. DHL92* |

**Table S4 The *CRY1* gene information in different plants**

| **Gene ID** | **Gene name** | **Strand** | **Gene position** | **CDS/bp** | **Proten/aa** | **MW/kD** | **pI** | **Subcellar location** |
| --- | --- | --- | --- | --- | --- | --- | --- | --- |
| AT4G08920 | *AtCRY1* | + | 5723650-5727268 | 2046 | 681 | 76.694 | 5.23 | nucleus |
| CsaV3_3G047490 | *CsCRY1* | + | 38763751-38768676 | 1839 | 612 | 69.366 | 5.46 | nucleus |
| MELO3C003575 | *CmCRY1* | + | 2265520-2269863 | 2046 | 681 | 76.609 | 5.41 | nucleus |
| CmoCh09G005460 | *CmoCRY1* | + | 2606252-2609725 | 2049 | 682 | 76.912 | 5.34 | nucleus |
| CmoCh01G016510 | *CmoCRY1* | + | 12513159-12517001 | 2052 | 683 | 76.992 | 5.28 | nucleus |
| Cla97C11G220070.2 | *ClaCRY1* | + | 26128139-26131961 | 2046 | 681 | 76.582 | 5.32 | nucleus |
| Moc11g06420.1 | *MocCRY1* | + | 4359455-4363079 | 2046 | 681 | 76.662 | 5.49 | nucleus |
| Bhi09M002062 | *BhiCRY1* | + | 66333821-66338958 | 2046 | 681 | 76.523 | 5.4 | nucleus |

**Supplementary Figure 1 The cucumber seedlings CR and CS growth under different light intensities of 10, 40, 80, 120, 160 umol m^-2^ s^-1^  for 9 days. The hypocotyl length, cotyledon length, and cotyledon width were measured daily.**

**
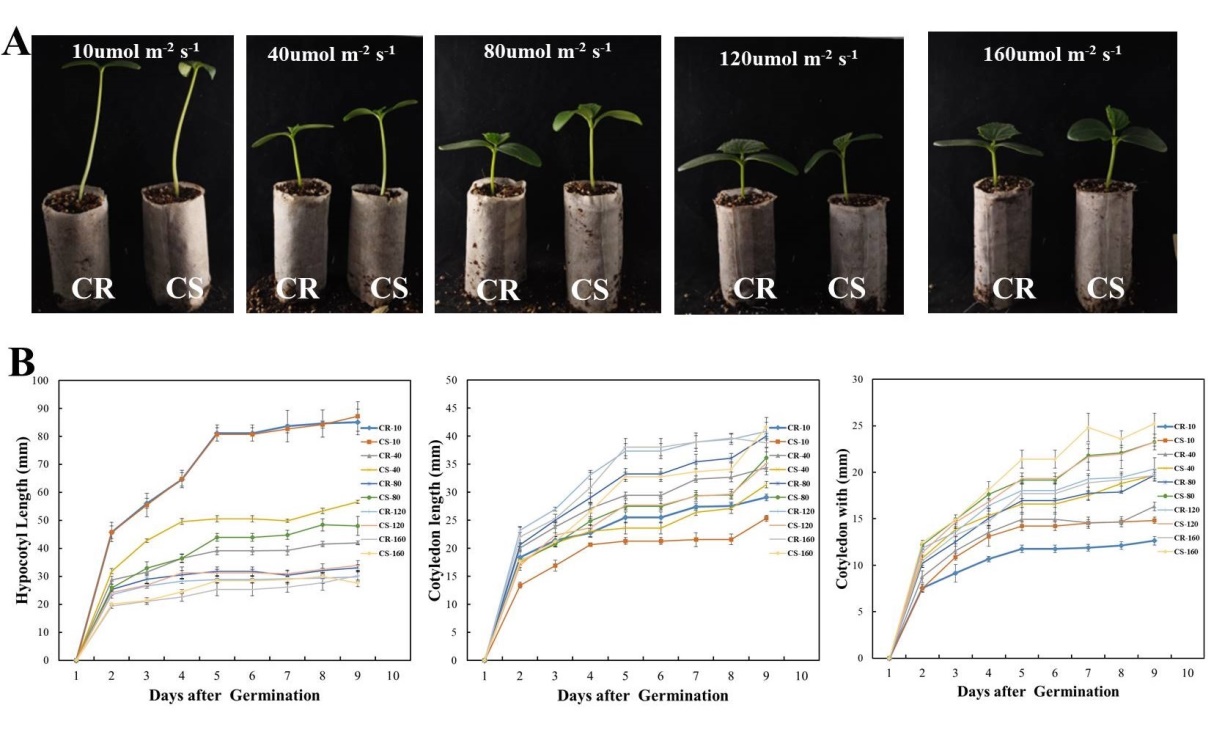
**

**Supplementary Figure 2 The SPAD and Fv/Fm value of CR and CS under low light stress condition. Here, the low case letters indicate significant differences at P < 0.05 by the least significant difference test.**

**
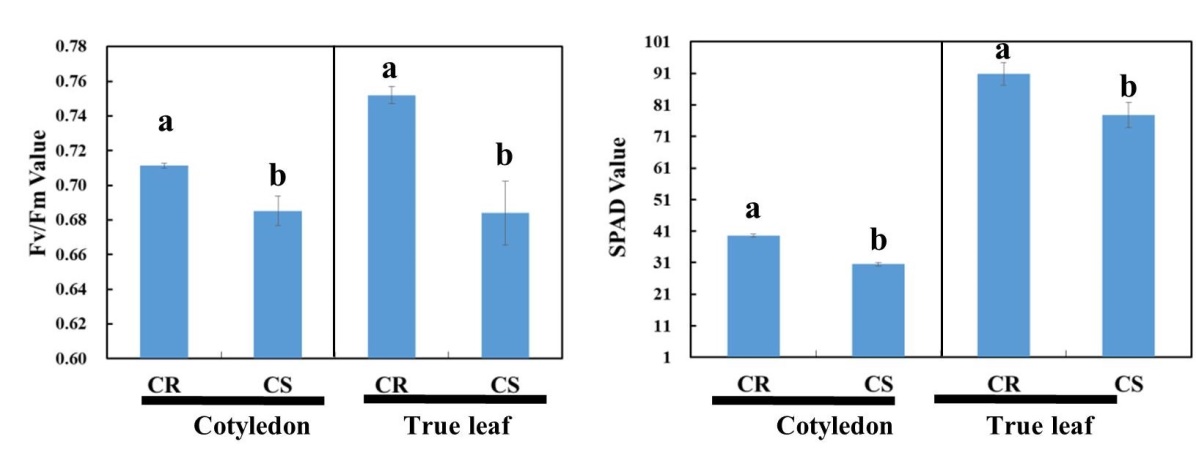
**

**Supplementary Figure 3 The hypocotyl length of cucumber seedlings (CR and CS) growth under different light quality treatment including Red light( 40 umol m^-2^ s^-1^ ), Blue light( 40 umol m^-2^ s^-1^ ), and Dark ( 0 umol m^-2^ s^-1^ ) for a week. Here, the low case letters indicate significant differences at P < 0.05 by the least significant difference test.**

**
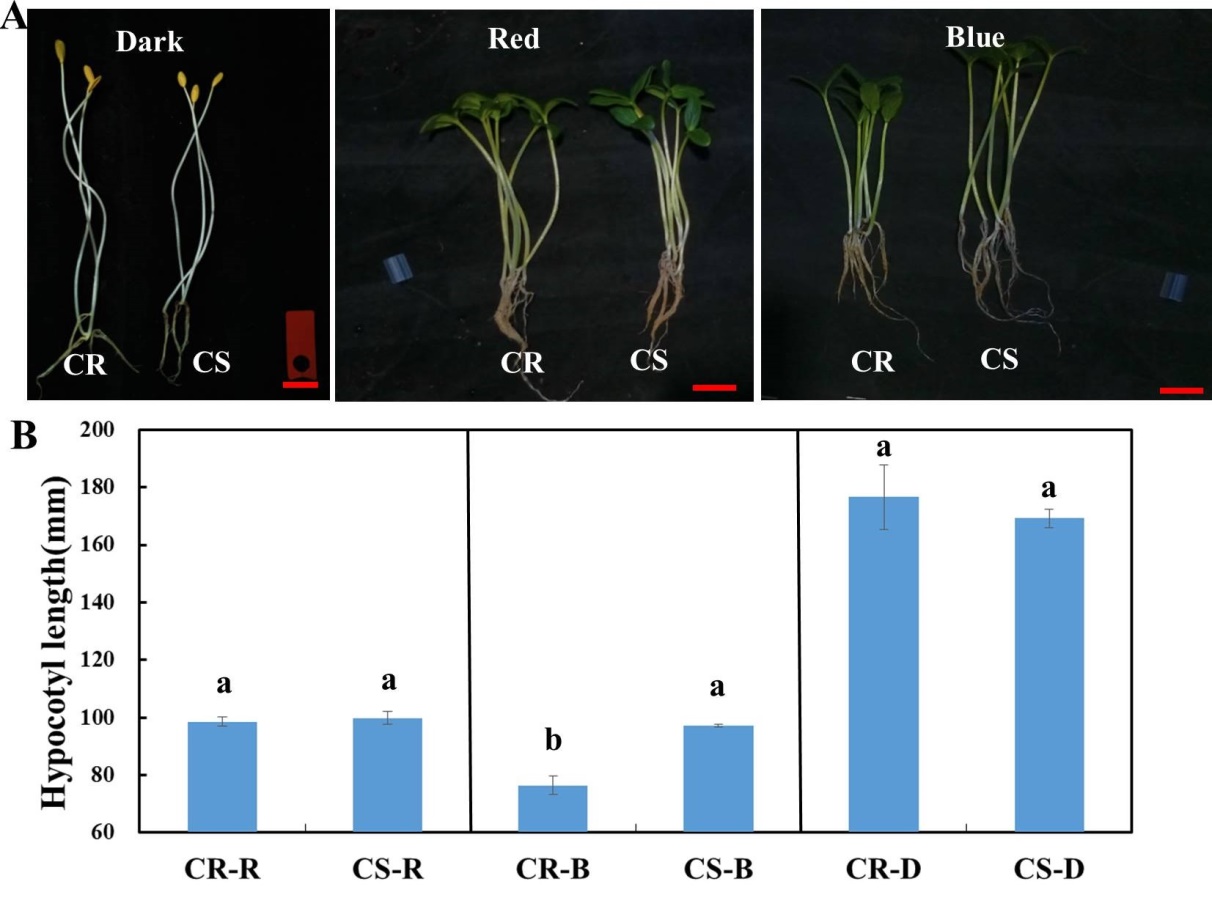
**

**Supplementary Figure 4 Supplement of different LED light qualities on the elongation of cucumber hypocotyl (CS) under low light stress. W40 represents 40umol m^-2^ s^-1^ low light stress; W40+R represents supplement of 120 umol m^-2^ s^-1^ red light under 40umol m^-2^ s^-1^ low light stress; W40+B represents supplement of 120 umol m^-2^ s^-1^ blue light under 40umol m^-2^ s^-1^ low light stress; W160 represents supplement of 120 umol m^-2^ s^-1^ white light under 40umol m^-2^ s^-1^ low light stress. Here, the low case letters indicate significant differences at P < 0.05 by the least significant difference test.
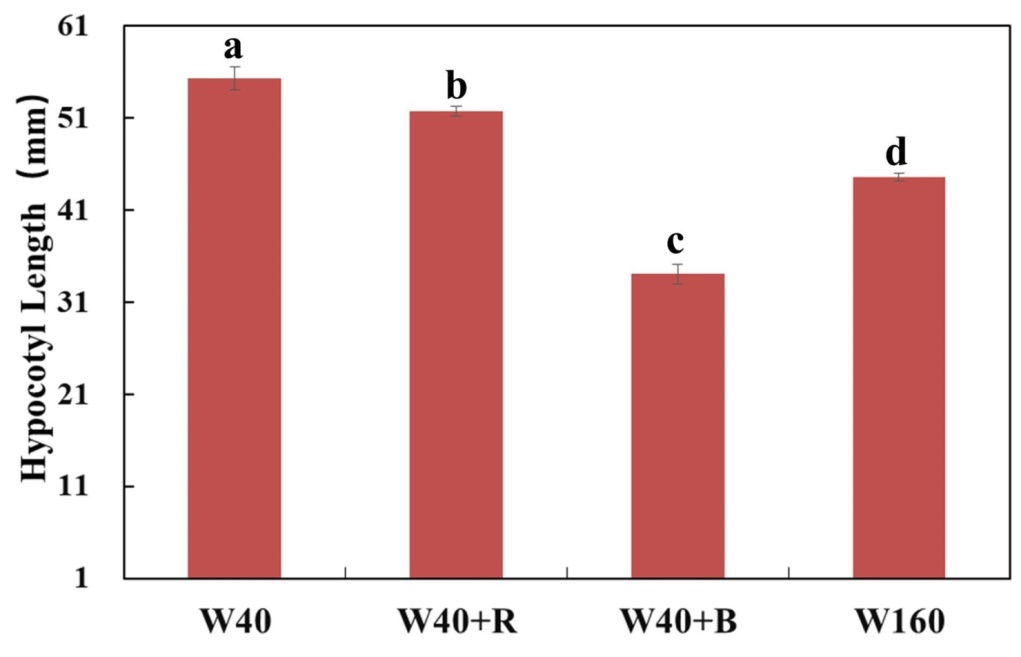
**

**Supplementary Figure 5 Heat map of photoreceptor genes in different tissue from bitter melon (A) and watermelon (B).**

**
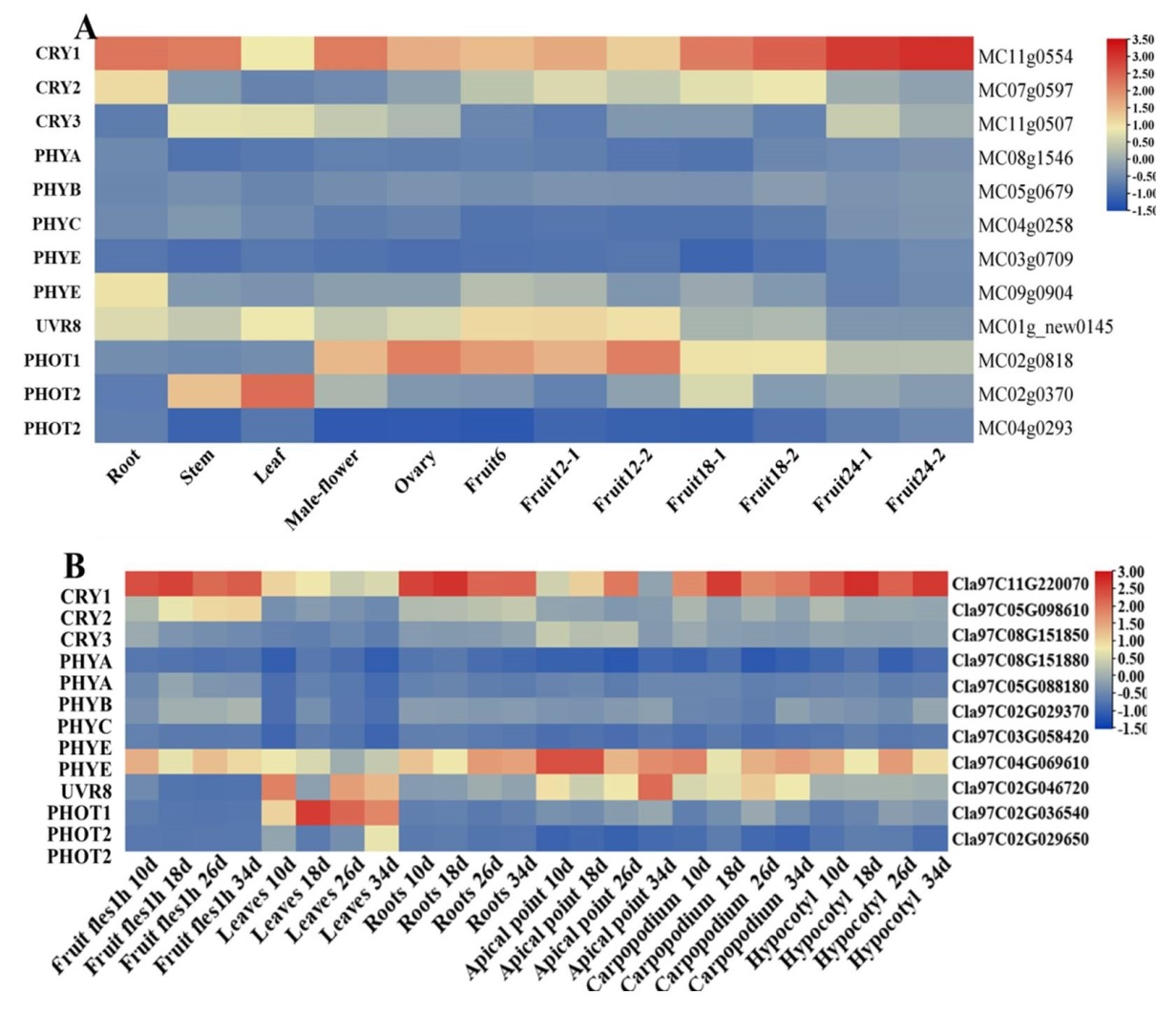
**

**Supplementary Figure 6 Cucumber hypocotyl (CS) length of CR and CR under 40umol m^-2^ s^-1^ low light stress and 160 umol m^-2^ s^-1^ white light control condition. Here, the low case letters indicate significant differences at P < 0.05 by the least significant difference test..**


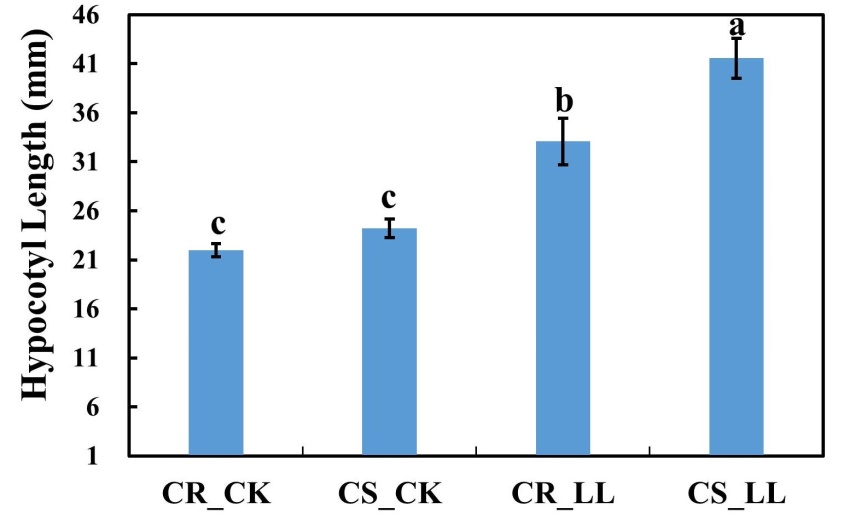


**Supplementary Figure 7 The transcripts structure of cucumber *CsCRY1*, obtained from single-molecule long-read sequencing.**

**
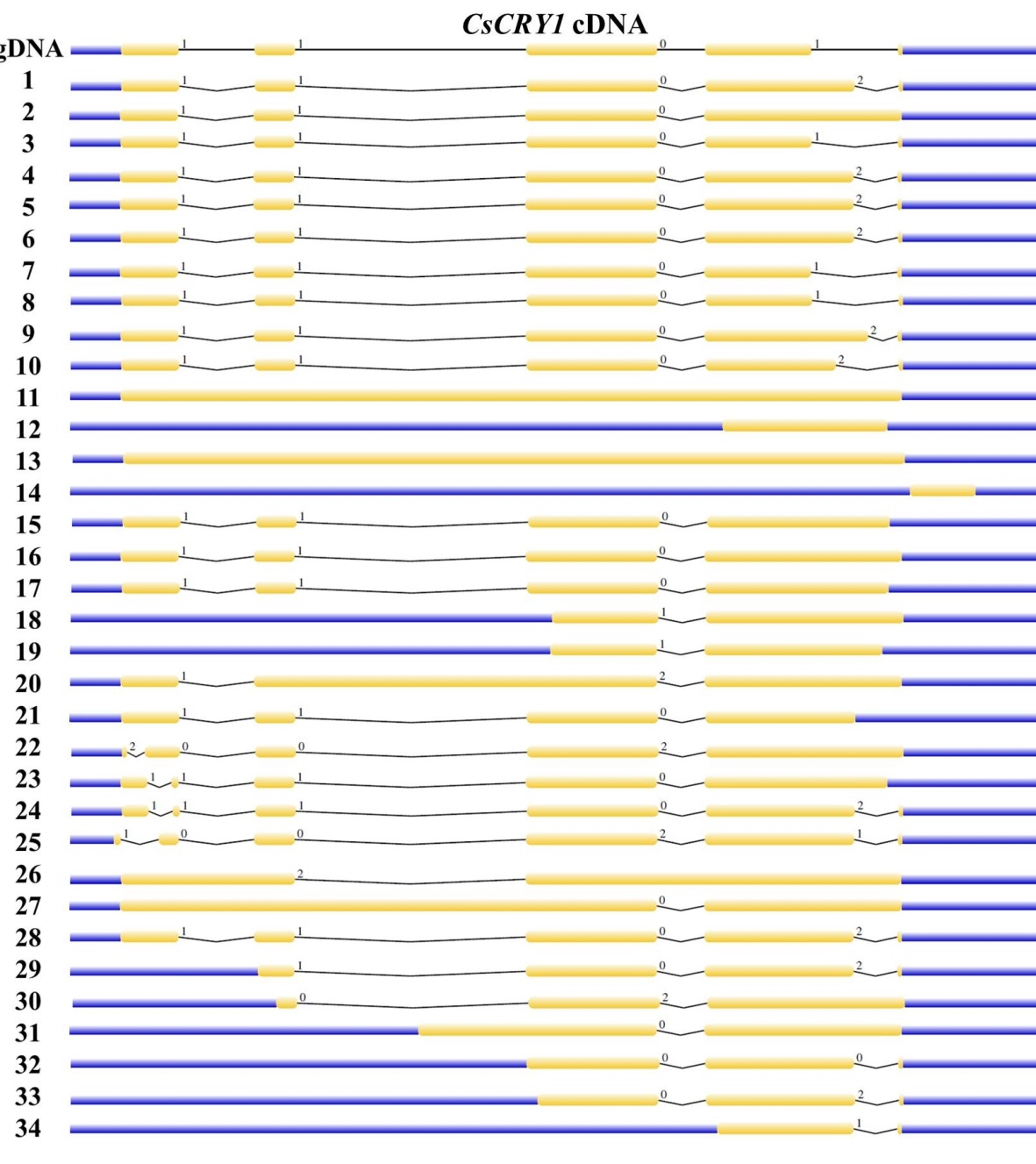
**

**Supplementary Figure 8 The transcripts read mapping results of CRY1 from *Cucumis melo L., Arabidopsis thaliana, Cucumis sativus L., and Cucurbita moschata.***

**
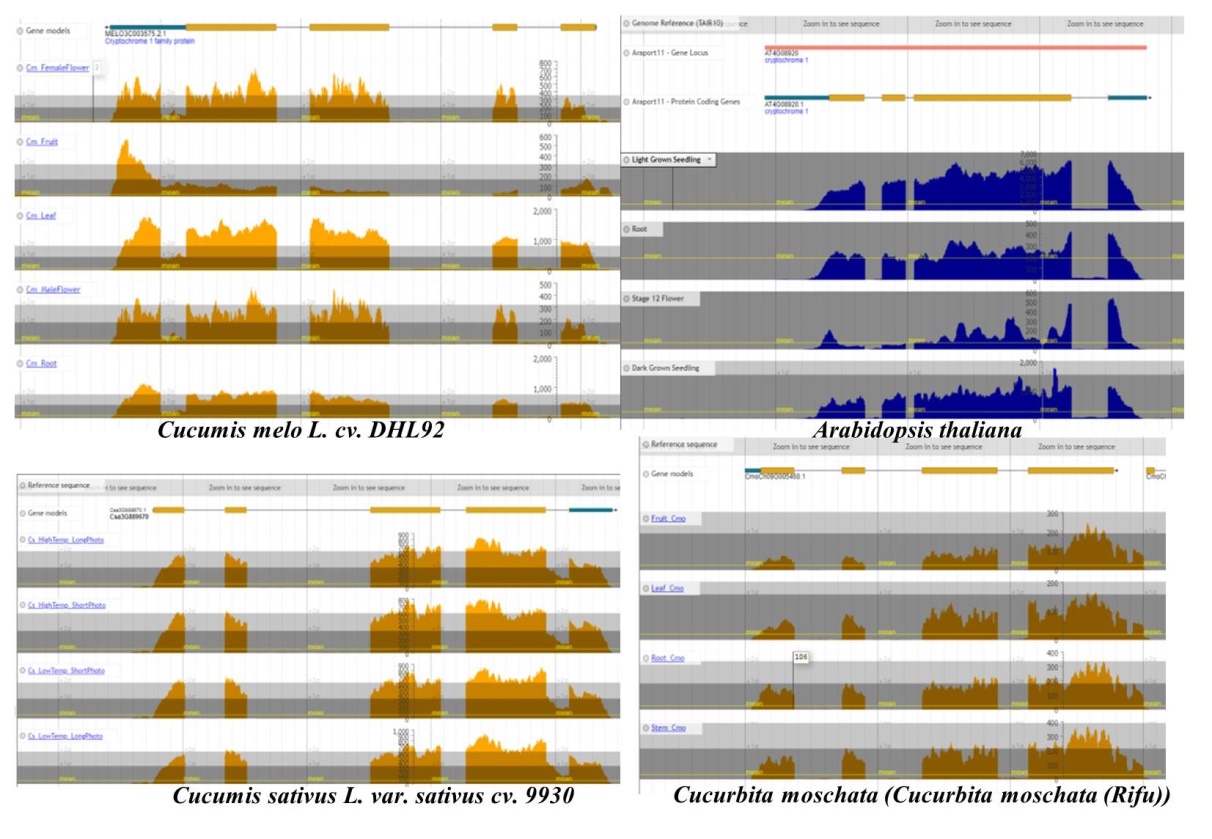
**

**Supplementary Figure 9 The transcripts read mapping of CsCRY1 in CR under control and low-light-stress condition**

**
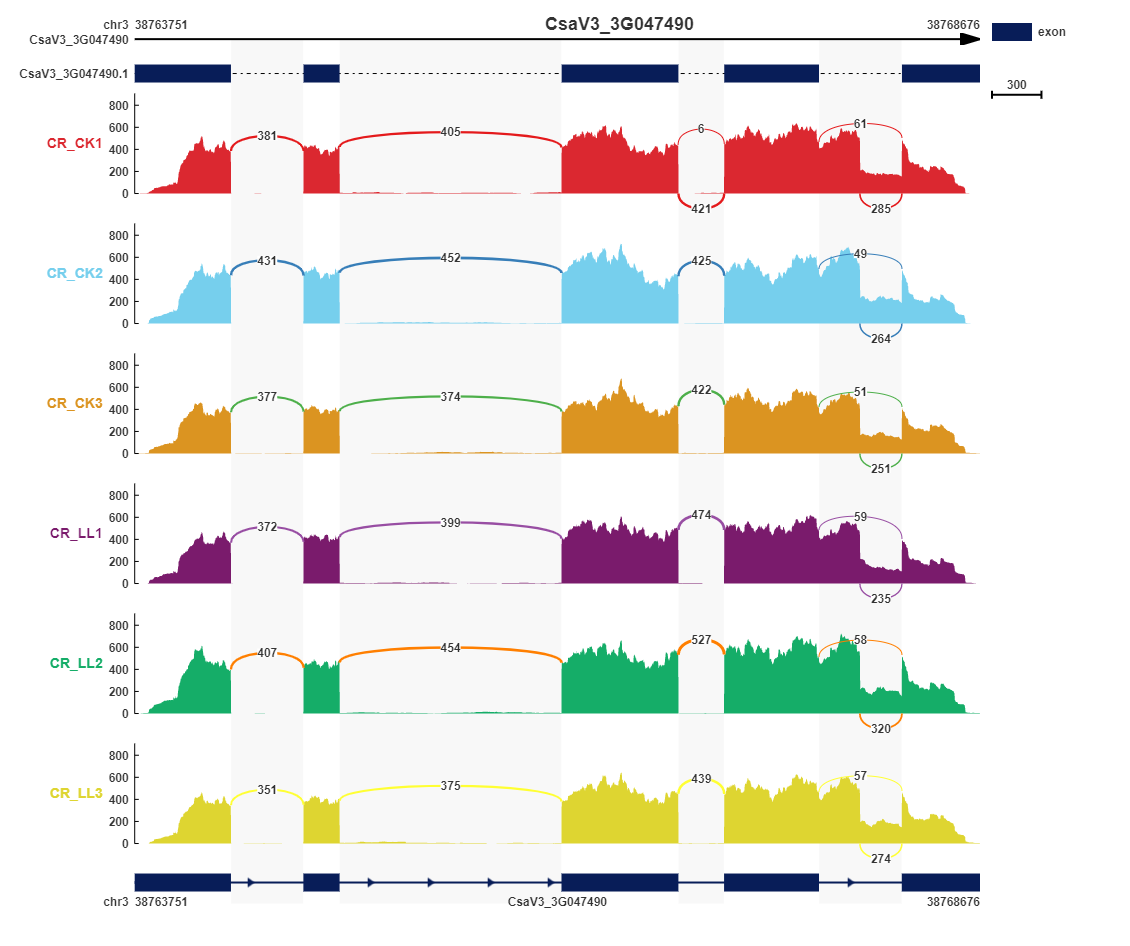
**
